# Acute Appendicitis in Patients with Kartagener Syndrome

**DOI:** 10.1155/2020/8716474

**Published:** 2020-02-27

**Authors:** Pedro Nogarotto Cembraneli, Gabriel Ambrogi, Julia Brasileiro de Faria Cavalcante, Raphael Raphe, Rafael Luís Luporini

**Affiliations:** ^1^Medical Sciences Course, Health Sciences School, Faculdade Ceres (FACERES), São José do Rio Preto, SP, Brazil; ^2^Medical Sciences Course, Health Sciences School, Universidade Metropolitana de Santos (UNIMES), Santos, SP, Brazil; ^3^Medical Sciences Course, Health Sciences School, Faculdade Ceres (FACERES), São José do Rio Preto, SP, Brazil; ^4^Department of General Surgery, Santa Casa de São Carlos, SP, Brazil; ^5^Department of Medicine at the Federal University of São Carlos (UFSCAR), SP, Brazil

## Abstract

Situs inversus totalis is a congenital syndrome characterized by a total left-right transposition of all abdominal and thoracic organs. It may be associated with chronic respiratory conditions such as sinusitis and bronchiectasis, composing the Kartagener syndrome. If not detected, this condition may compromise the early diagnosis of surgical emergencies such as cholecystitis and appendicitis. A rare case of appendicitis in a patient with Kartagener syndrome is here reported.

## 1. Introduction

Primary ciliary dyskinesia (PCD), an autosomal recessive genetic condition, has been widely described worldwide. Its prevalence is 1 in 10,000–30,000 individuals of both sexes. About 50% of the patients with PCD have situs inversus totalis (SIT) [1, 2], a rare anomaly defined by a total left-right transposition of all abdominal and thoracic organs, i.e., the organs are located in the opposite side of their normal positions. It is estimated that the prevalence of SIT varies between 1 in 5,000 and 1 in 20,000 live births [3]. Individuals presenting with the triad SIT, chronic sinusitis, and bronchiectasis have the rare Kartagener syndrome (KS) [1, 2], a type of PCD with an estimated prevalence of 1 in 60,000 individuals [4].

The fact that a patient has SIT can delay the diagnosis in cases of surgical emergencies [5]. Acute appendicitis is one of the main causes of acute abdomen that requires a surgical procedure, and its diagnosis is based on patient's symptoms and clinical signs. Most of the patients report the classical symptomatology, but in case of doubt, the surgeon can order laboratory tests and imaging exams to support the diagnosis [6, 7]. We report a rare case of appendicitis in a patient with KS.

## 2. Case Report

A 29-year-old male arrived at the emergency room complaining of epigastric abdominal pain. He reported that the sudden onset of the pain had started a few hours earlier, with radiation to the left iliac fossa. In association with this symptom, he had nausea, but denied vomiting and hyporexia. The frequency of bowel evacuation was 7 times in 24 hours, with Bristol Stool Form Scale 6–7. The patient had a personal history of allergic bronchitis since childhood and chronic sinusitis.

**Figure 1:**
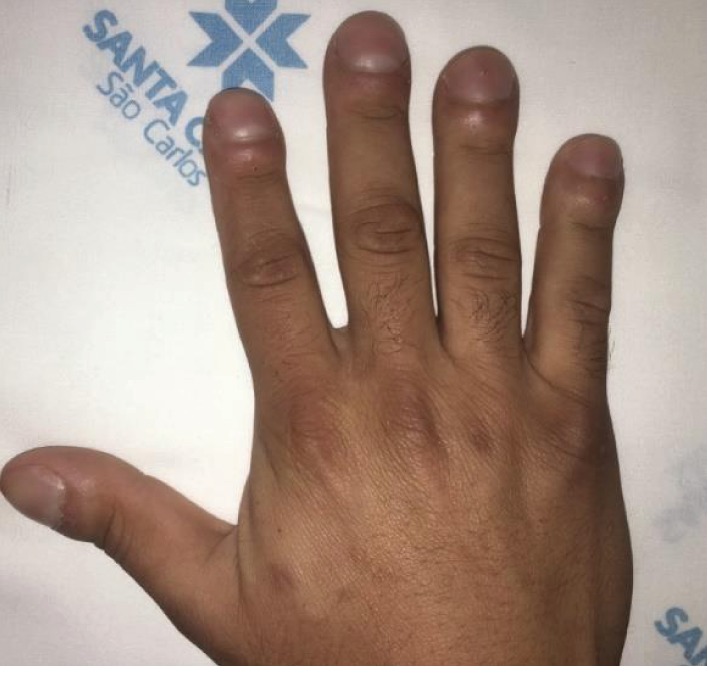
Digital clubbing.

At physical examination, he was conscious, with no signs of hemodynamic instability. The inspection revealed digital clubbing (Figure 1), a sign of PCD. Pulmonary auscultation showed no alterations, whereas heart auscultation detected ictus cordis to the right, and the classic auscultation points were also situated in the right hemithorax. Abdominal palpation confirmed that the patient had pain in the left iliac fossa, but no signs of peritonitis were found.

**Figure 2:**
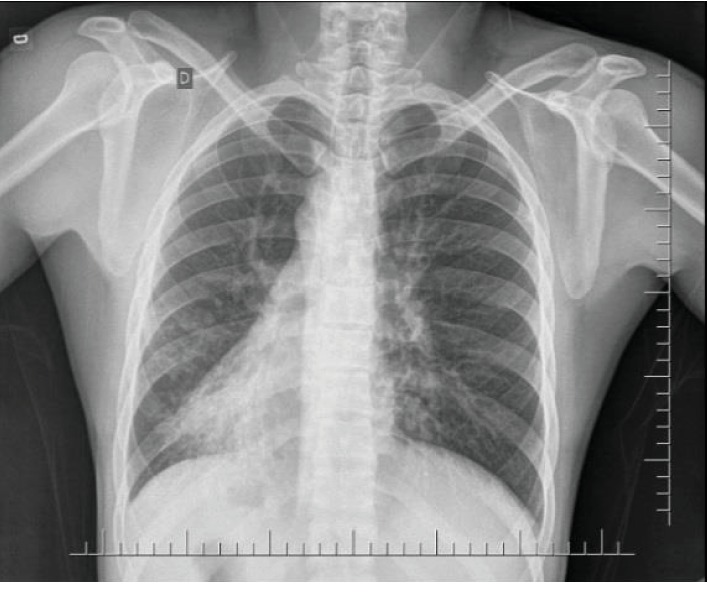
Chest X-ray showing dextrocardia.

**Figure 3:**
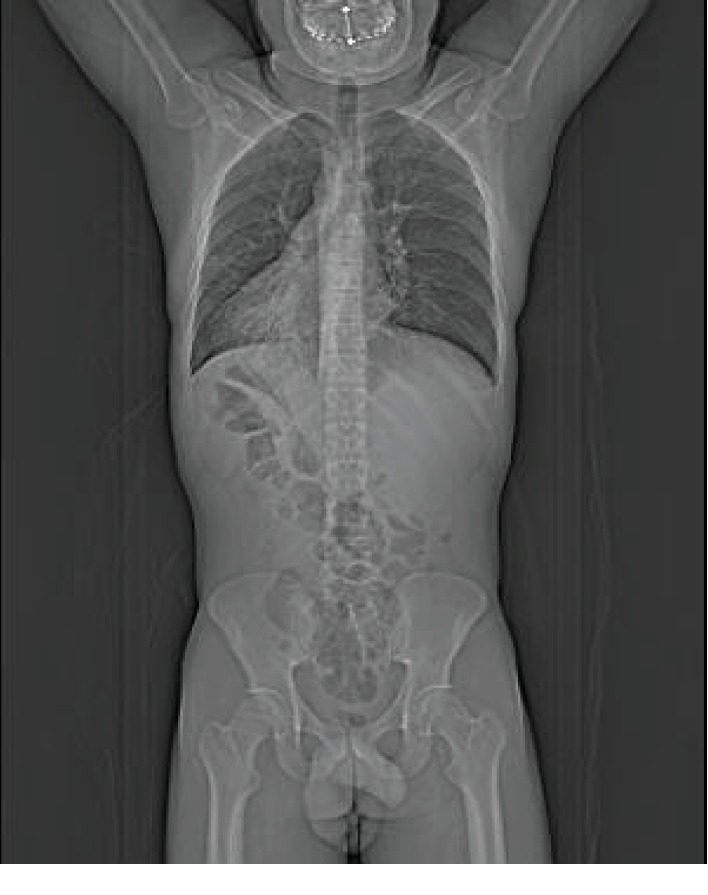
Multiplanar reconstruction in computed tomography scan highlighting the transposition of the great vessels and the abdominal and thoracic organs.

**Figure 4:**
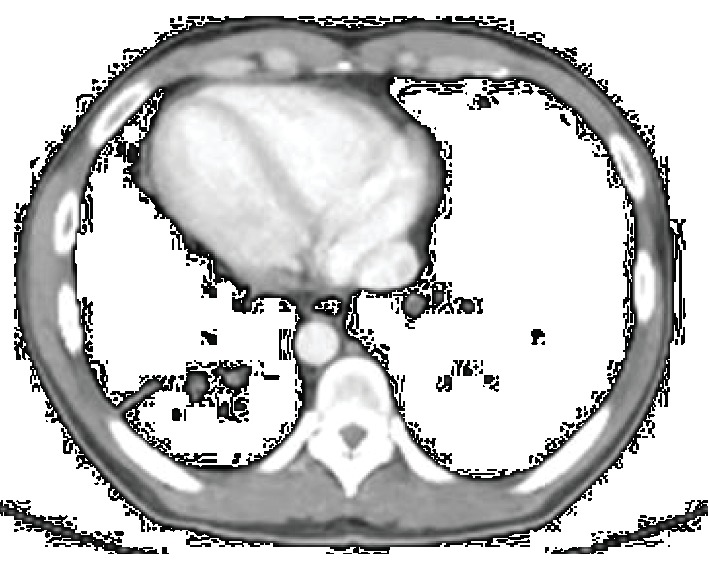
Computed tomography scan axial cut in the mediastinal window showing dextrocardia and bronchiectasis.

Laboratory tests were requested and the results were leukocyte count 19,000/mm^3^ with 5% rods in the blood (leukocytosis), 28,000 leukocytes/mL in the urine (leukocyturia), and C-reactive protein 8.90 mg/dL. Abdominal and chest X-rays were performed and showed dextrocardia (Figure 2). Computed tomography (CT) scans of the thorax and abdomen were done, revealing SIT, characterized by the transposition of the great vessels as well as of the abdominal and thoracic organs (Figure 3). Signs of bronchiectasis, bronchiectasis, thickening of the bronchial and bronchiolar walls, and mucoid impaction were also visualized in the imaging exams (Figure 4).

**Figure 5:**
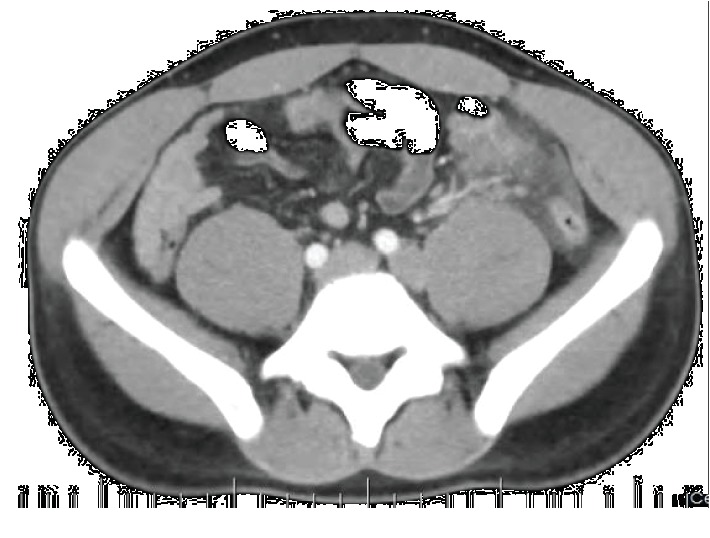
Computed tomography scan axial cut evidencing blurring view of the adjacent visceral fat in the left iliac fossa.

Evidence of wall thickening was found in the colon, associated with mucosal edema and blurring view of the adjacent visceral fat in the left iliac fossa (Figure 5). Based on the symptoms and clinical signs, laboratory tests, and imaging exams, the patient was diagnosed with acute appendicitis on the left side, and the surgical intervention was carried out.

**Figure 6:**
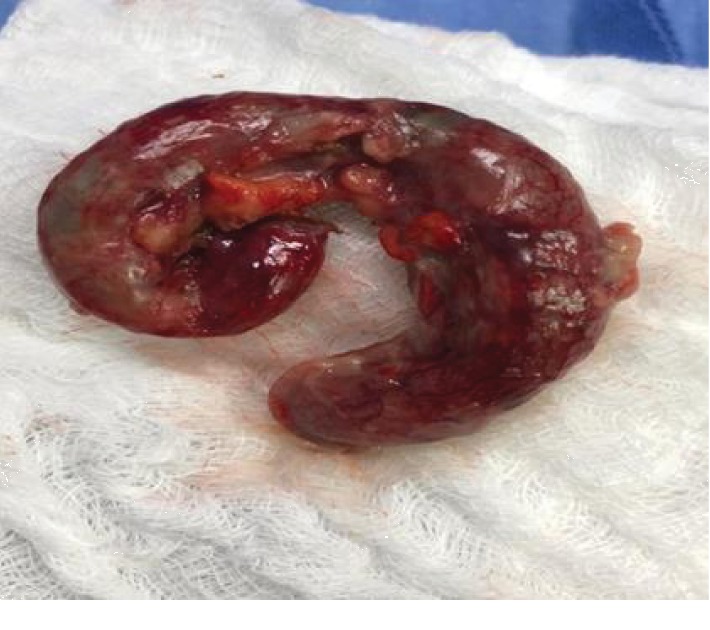
Grade 3 appendicitis (perforated with localized free fluid) removed from the patient.

**Figure 7:**
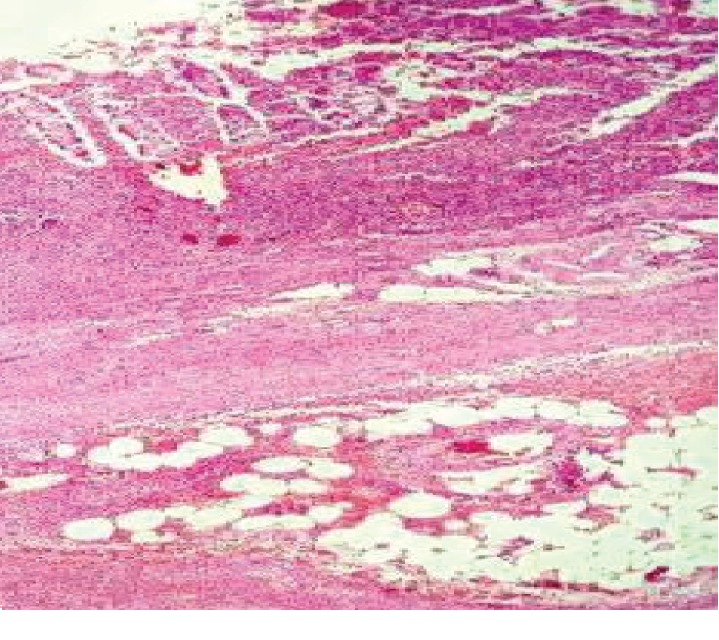
Histological section at 40x magnification showing fibrinopurulent exudate in all the layers of the appendix. The muscle layer of the appendix diffusely infiltrated with abundance of neutrophils confirms the diagnosis of acute appendicitis.

An oblique incision was made on the left lower quadrant of the abdomen, with dissection through the anatomic planes to open the peritoneal cavity. After identifying the cecal appendix, showing grade 3 appendicitis (Figure 6), ligation of the mesoappendix and pouch suture at the base of the cecum were performed. The appendectomy was carried out after this double ligation, followed by invagination of the appendicular stump, thorough inspection of the peritoneal cavity, washing the area with sterile fluid, closing the incision, and dressing the surgical wound. The anatomical pathology examination confirmed the diagnosis (Figure 7).

In addition to the need of the surgical procedure, the diagnosis of KS was suggested by the clinical history and the imaging exams. In the postoperative period, the extubation of the patient was difficult due to bronchiectasis. Despite this complication, his evolution was good, and he stayed in the ward for 48 hours before being discharged by the general surgery team. Subsequently, he was referred to the outpatient clinic for follow-up. He did not have any long-term complications related to the surgical procedure, and he currently has good clinical control of the symptoms of KS.

## 3. Discussion

Appendicitis is an acute inflammation of the vermiform appendix, most likely due to luminal obstruction by a fecalith and/or lymphoid hyperplasia, among other possibilities [8]. Typically, the patient describes anorexia, acute ill-defined abdominal pain, beginning in the mesogastric region and then moving to the right iliac fossa, associated with fever, nausea, and vomiting [9]. Since pain located in the left iliac fossa resulting from acute appendicitis is extremely rare, it may be related to congenital abnormalities such as intestinal malrotation or SIT [10]. This symptom may delay the diagnosis, because other possibilities are more likely diverticulitis, renal colic, ruptured ovarian cyst, Meckel's diverticulitis, epididymitis, prostatitis, intestinal obstruction, incarcerated hernia, enteritis, pelvic inflammatory disease, and mesenteric ischemia [11]. A delayed diagnosis increases the risk of complications such as perforation [12].

PCD is characterized by impairment of mucociliary clearance, predisposing the patient to chronic respiratory infections [5]. Dyskinesia occurs in all the structures lined by ciliated epithelium such as the nasal cavities, paranasal sinuses, middle ear, tracheobronchial tree, ependyma, efferent conduits, fallopian tube, endometrium, and retinal cells [13].

Abnormal ciliary motility leads to recurrent paranasal, sinus, and pulmonary infections, as well as to infertility due to impaired sperm motility. Furthermore, poor ciliary motility during embryogenesis predisposes to laterality defects such as SIT or dextrocardia with situs solitus [14].

Although KS is rare, health professionals should be aware of its characterization, mainly in patients with a surgical emergency, because of its atypical clinical presentation, which may lead to misdiagnoses. A complete anamnesis and an attentive physical examination are important to detect this underlying condition [15]. The occurrence of appendicitis associated with SIT poses a diagnostic challenge, especially in acute cases [16]. Therefore, appendicitis should be among the differential diagnoses of pain in the left iliac fossa during emergency care. Imaging exams such as CT, ultrasonography, thoracic X-ray, and diagnostic laparoscopy are of paramount importance for the early diagnosis of appendicitis and can help avoid unfavorable outcomes [17].
